# Neural Consequences of Chronic Short Sleep: Reversible or Lasting?

**DOI:** 10.3389/fneur.2017.00235

**Published:** 2017-05-31

**Authors:** Zhengqing Zhao, Xiangxiang Zhao, Sigrid C. Veasey

**Affiliations:** ^1^Department of Neurology, Changzheng Hospital, Second Military Medical University, Shanghai, China; ^2^Center for Sleep and Circadian Neurobiology, Department of Medicine, Perelman School of Medicine, University of Pennsylvania, Philadelphia, PA, United States

**Keywords:** locus coeruleus, sleep deprivation, neurodegeneration, vigilance performance, developmental biology

## Abstract

Approximately one-third of adolescents and adults in developed countries regularly experience insufficient sleep across the school and/or work week interspersed with weekend catch up sleep. This common practice of weekend recovery sleep reduces subjective sleepiness, yet recent studies demonstrate that one weekend of recovery sleep may not be sufficient in all persons to fully reverse all neurobehavioral impairments observed with chronic sleep loss, particularly vigilance. Moreover, recent studies in animal models demonstrate persistent injury to and loss of specific neuron types in response to chronic short sleep (CSS) with lasting effects on sleep/wake patterns. Here, we provide a comprehensive review of the effects of chronic sleep disruption on neurobehavioral performance and injury to neurons, astrocytes, microglia, and oligodendrocytes and discuss what is known and what is not yet established for reversibility of neural injury. Recent neurobehavioral findings in humans are integrated with animal model research examining long-term consequences of sleep loss on neurobehavioral performance, brain development, neurogenesis, neurodegeneration, and connectivity. While it is now clear that recovery of vigilance following short sleep requires longer than one weekend, less is known of the impact of CSS on cognitive function, mood, and brain health long term. From work performed in animal models, CSS in the young adult and short-term sleep loss in critical developmental windows can have lasting detrimental effects on neurobehavioral performance.

## Chronic Short Sleep (CSS) in Humans is Commonly Observed

Chronic short sleep, defined as frequently obtaining ≤6 h sleep/24 h period, is highly prevalent in both adults and adolescents. In the United States, CSS is reported by 30% of employed adults (night and day shifts) or over 40 million individuals (1). The CSS definition cutoff of 6 h may underestimate the number of people regularly experiencing insufficient sleep. Specifically, an in-lab study of healthy young adult humans examining the cumulative effects of 4–10 h of sleep on vigilance performance determined with mathematical modeling that >8 h of sleep/night is needed to prevent decrements in vigilance (2). College students are also likely to experience CSS. A poll conducted among college students found that 25% of the students regularly obtained <6.5 h of sleep and 70% obtained <8 h/night (3). A poll obtained in the United States found that 97% of 12th grade students report less than the 9 h of sleep per night recommended for teenagers, and 75% reported regularly obtaining <8 h/night (4). There are intriguing geographical and/or cultural variations in reported sleep time. Within the United States, the region with the highest percentage of individuals reporting regularly obtaining insufficient sleep is within the mid-Atlantic Appalachian mountains (5). In South Korea, the mean reported sleep time for teenagers in one large study was 4.9 h/night (6). In contrast, Australian adolescents average 8.5–9.1 h/night and less commonly sleep in over weekends (7). Caffeine use, electronic devices in bedrooms, and school start times have all been shown to influence total sleep times for adolescents (8). Delaying school start time by 1 h in the United States increases sleep times in high school students and reduces both sleepiness and the incidence of student motor vehicle accidents (9–11), suggesting that circadian phase delay contributes at least in part to the CSS in American adolescents. In summary, high percentages of the workforce, college and high school students regularly experience CSS, where both cultural and biologic factors contribute to the high prevalence of CSS. While cardiovascular and metabolic effects of CSS have been substantiated (12–14), we are only beginning to explore the lasting neurobehavioral consequences of insufficient sleep. At present, we do not know whether the shorter sleep times have lasting effects on neurobehavioral performance and/or brain aging.

To try to understand needed sleep times, several recent studies have examined sleep times in adults living in pre-industrialized societies (15–17). One study compared two regionally proximal populations, one with and one without electricity, found that those with access to artificial light slept almost 1 h less (16). However, average sleep durations for three distinct groups of hunter-gatherers each on a different continent had total sleep times of 6–7 h (17), which is not different from average sleep times in developed countries. None of these studies, however, addressed the question how much sleep is needed for optimal performance. It is interesting that in controlled laboratory polysomnography studies in developed societies, sleep times in most individuals allowed 9 h time in bed were over 8 h (2, 18), suggesting that sleep needs may vary across developed and undeveloped societies, more than geographically. It is also possible that individuals are limited by wakefulness activities and duration, to a larger extent than sleep duration (2).

## Protracted Recovery of Neurobehavioral Impairment Following CSS in Humans

Contrary to the widely appreciated subjective normalization of sleepiness after a weekend of recovery sleep, several studies have now shown clearly that shortened sleep across 1 week in healthy adults produces cumulative impairments in vigilance with incomplete recovery after three full nights of recovery sleep (2, 18, 19). Unfortunately in each of the studies examining recovery after partial sleep loss across the week, the recovery was not extended long enough to observe full recovery. Subjective improvement in sleepiness is greater than objective improvement, which may help explain why the pattern is repeated week after week in so many individuals (20). Incomplete recovery in vigilance raises the possibility of lasting injury or neuronal dysfunction from CSS. While vigilance remains impaired after three nights of recovery sleep, performance with other tasks sensitive to sleep loss, such as a visual-motor task, normalizes after the second night of recovery sleep (21). In adolescents, sleep architecture after seven nights of sleep restriction (5 h sleep) remained abnormal after three nights of recovery sleep, with greater sleep efficiency and a shorter latency to non-rapid eye movement (NREM) stage 2 sleep (22). Moreover, neurobehavioral performance in adolescents may be more vulnerable to sleep restriction than in adults. When adolescents are restricted to 4 h sleep per night for seven nights, processing speed remained slowed even after two full recovery nights of sleep (23). Collectively, these findings support: (1) recovery requiring >3 nights for specific neurobehavioral performances; (2) a differential susceptibility to CSS across various tasks, where vigilance is particularly sensitive to CSS; (3) a differential susceptibility for age in that adolescents may require longer to recover from CSS neurobehavioral impairments; and (4) recovery of circuits integral to behavioral state and/or vigilance is delayed following CSS.

## Animal Models of Sleep Loss

Various sleep disruption protocols have been implemented in animal research. Each model has its own set of benefits, limitations, and addressable sleep research questions as well as its own set of potentially confounding variables. Because of its simplicity and ease to administer, many chronic sleep loss studies have used permutations of the platform-over-water technique, in which animals must stay awake and maintain balance on a small platform to avoid falling into a water tray (24–28). The platforms can be sized to minimize primarily rapid eye movement sleep (REMS) or both non-rapid eye movement sleep (NREMS) and REMS. In earlier studies, animals were singly housed. This particular paradigm, however, causes significant stress for most rodents (28). More recently, chambers with multiple platforms have been utilized, that allow group housing (29). Nonetheless, animals cannot nest or sleep next to one another, and animals slept deprived by the platform method tend to lose weight, suggesting some influence on metabolism or stress (29). Several sleep deprivation apparatuses have been developed to enforce locomotion to maintain wakefulness. These devices have a sweeping disk, treadmill, or bar that prods a sleeping animal to move (30). This technique is also highly effective in inducing sleep fragmentation, but may not prevent all sleep (31). In fact, clever animals can learn to sleep upon the sweeping bar. The forced activity devices require single housing and animals will have microsleeps, between times when the bar or disk prods the animal (31). Mice also may be kept awake by gentle handling which allows mice to remain in their home cages with cage mates (32, 33). The gentle handling method is labor intensive, requiring a researcher to monitor animals continuously and provide a tactile stimulus when animals are still. An additional method effectively implemented for chronic sleep loss is environmental enrichment (34, 35). Researchers have used this technique to maintain continuous active wakefulness for 3–8 h/day across repeated days, modeling sleep patterns in shift workers or students pulling late study nights (35, 36). After the initial exposure, animals exposed to this model have normal corticosterone levels to suggest no major effects on stress (35). Moreover, animals exposed to enriched environment sleep loss do not lose weight across the paradigm. The animals catch up on some of the missed sleep across the undisturbed dark period, so that overall, the animals lose approximately 4 h of sleep in a 24-h period (35). This method of chronic sleep loss is labor intensive, requiring a researcher to be present at all times to monitor animals and add new bedding, toys, etc. when mice are still. This particular method is most appropriate when examining the effects of extended exploratory wakefulness, as mice can be effectively kept awake with minimal microsleeps for at least 8 h. In summary, the implemented sleep loss paradigms vary by stress induced, metabolic effects, locomotor effects, microsleeps, and intermittent sleep periods. Newer methods of sleep loss, including optogenetic and chemical genetic long-term stimulation of wake-activating neurons to induce extended wakefulness and sleep fragmentation, are being optimized for long-term effectiveness. Pharmacogenetics would allow non-invasive enforced wakefulness in group housed mice, while optogenetics is expected to offer a valuable window into the molecular responses in wake neurons to various levels and durations of activation of specific groups of neurons. Very few paradigms have been compared within a lab or across species to provide insight into species differences and sleep deprivation models differences in neural effects. Table 1 summarizes approaches, species, and neural findings for experiments.

**Table 1:** **Neural effects of sleep disruption across various experimental models and species**.

Sleep loss paradigm	Duration	Animals	Neural findings
Small platform	2 weeks	Adult rats	No differences in apoptosis markers (whole brain) or stress genes (cerebral cortex) in sleep-deprived and yoke controls (37)
Enriched environment	8 h/day 3 days/week	Adult mice	Loss of locus coerueus and orexinergic neurons (35, 36)
Rotating drum	3 h on/1 h off 100–150 h	Adult rats	Reversible and non-progressive vigilance impairment (38)
Small platform	20 h/day for 5 days	Adult rats	Intact homeostasis (39)
Sleep fragmentation (rotor table)	1 arousal/2 min 14 weeks	Adult mice	Wake neuron degeneration and reduced c-fos activation (40)
Sleep fragmentation (sweeper bar)	1 arousal/2 min 2–7 weeks	Adult mice	Leptin resistance, hypothalamic endoplasmic reticulum stress (41); hippocampal learning and memory impairments and inflammation (42, 43)
Gentle handling when still	12–36 h	Adult rats	Neuronal chromatolysis and vacuolization in cortices >locus coeruleus (44)
Rotating drum	20 h/day for 8 weeks	Adult mice	Impaired hippocampal learning and memory; increased cortical amyloid β peptides (45)
Enriched environment and caffeine	6–8 h for 4 days	Adolescent mice (1 month)	Pyramidal neurons: increased lysosomes and mitochondrial injury in frontal cortex (46)
Small platform	7 days	Young rats (3 weeks)	Impaired long-term potentiation in visual cortex (47)
Gentle handling and auditory stimulation	12 h	Young cats (3–4 weeks)	Impaired visual cortical plasticity
Vial rocker	24 h	Young flies (1 day)	Long-term change in dopamine receptors and memory impairments that were rescued with dopamine agonists (48)
Vial rocker	24 h	Young flies (1 day)	Impaired reproductive behavior as an adult; developmental injury to olfactory glomerulus (49)
Gentle stimulation, enriched environment and rotating platform	70% sleep loss for 4 days	Adolescent mice (1 month)	Increased synaptic contact by astrocytes (50)

## Neural Findings in the Rechtschaffen Rat Studies of Sleep Deprivation

In landmark studies in the late 1970s and early 1980s, Allan Rechtschaffen and collaborators showed that continuous sleep deprivation in adult rats, using singly housed disk over water methodology, resulted in death of the animals within 2–4 weeks (51). While the cause of death was not evident in all cases, all rats lost tremendous amounts of weight and developed both malnutrition (protein wasting) and chronic skin ulcerations. Many of the animals succumbed to bacterial sepsis. Remarkably, rats on the brink of death from total sleep deprivation could experience what appeared to be a complete physiological recovery if allowed 2 weeks of uninterrupted recovery sleep (52). Despite the prominent peripheral physiological effects from total sleep deprivation, brains of rats exposed to prolonged sleep deprivation did not evidence obvious injury. Specifically, there was no increase in apoptosis or other neural markers of degeneration when examining 6–10 sections throughout the brain for all cell types (37). If animals exposed to this severe sleep loss do not incur obvious neurodegeneration, should we expect injury with the less severe chronic sleep restriction, as experienced by humans? Several important considerations are necessary to most accurately address this question. First, sleep-deprived animals in this platform paradigm were compared to yoked control animals that were also exposed to single housing on a platform over water (stressful for rodents) where some sleep loss and sleep fragmentation also occurred in these yoked controls. Thus, it is possible that less severe sleep loss in controls and severe sleep loss cause comparable injuries. Second, accurate determination of neurodegeneration requires performing neuronal stereology; other less robust measures may lead to inaccurate conclusions. Third, if only small subsets of specific neurons are injured, examining neurons collectively might obscure differences, and fourth, finding normal cell counts does not ensure normal brain function. The brain relies on connectivity and neuronal responsiveness to function well. For these reasons, it is possible that less severe chronic sleep loss could injure and even kill select populations of neurons in the brain, impair functionality and/or connectivity.

## Lasting Effects of CSS on Wake-Active Neurons

The incomplete cognitive recovery in vigilance observed in humans raises the possibility of lasting injury from CSS. Within the brain and brainstem are specific populations of neurons that are activated across wakefulness, quiescent only during sleep (53). One of these groups of neurons, the brainstem locus coeruleus neurons (LCn) is a collection of neurons essential for vigilance (54, 55). If sleep loss injures LCn from extended wakefulness, the injury should be greater for longer durations of wakefulness, and, indeed, LCn respond to short sleep loss (one period of 3 h of sleep loss) with an upregulation of antioxidant enzymes (35). With extended sleep loss (modeling 8 h of sleep loss on three consecutive days, followed by catch up sleep opportunities during the normal active period), there is no upregulation of antioxidant enzymes, and importantly, the neurons evidence increased reactive oxygen species (35). Sirtuins are deacetylase enzymes that play key roles in cellular homeostasis. The major mitochondrial sirtuin is sirtuin type 3 (SirT3) (56). Short-term sleep loss resulted in LC neuron upregulated SirT3, which then activates FOXO3a leading to antioxidant transcription (35). Importantly, in short-term sleep loss, SirT3 was essential for the adaptive upregulation of antioxidants and maintenance of redox homeostasis in LC neurons, in that mice lacking SirT3 did not upregulate antioxidants and consequently accrued superoxide in the LC across just 3 h of wake (35). The adaptive SirT3-dependent response, however, was short-lived. Upon extended sleep loss, 8 h sleep loss on three consecutive days, the SirT3 response failed, as evidenced by mitochondrial protein hyperacetylation (35); LC neurons did not upregulate antioxidant enzymes, and LC neurons succumbed to severe oxidative stress including the accumulation of lipofuscin, indicative of mitochondrial injury and senescence. Most importantly, extended wake resulted in a 25–30% loss in LC neurons, demonstrating for the first time that CSS can result in neuron loss (35). Recently, we extended the duration of CSS to 4 weeks in wild-type mice to ascertain whether longer CSS exposures increase injury further, to identify other groups of neurons might be injured and to examine reversibility (36). LC neuron loss was greater (40%) with 4 weeks CSS and did not recover with 1 month of rest, supporting irreversible loss of neurons rather than down regulation of noradrenergic specific markers (36). In addition to a loss of LC neurons, orexinergic neurons (another wake-activated group of neurons) were also lost in part (40%), while a sleep-active group of neurons, the melanin-concentrating hormone neurons, adjacent to orexinergic neurons in the hypothalamus, conferred resistance to CSS neuron loss (36). Importantly, CSS results in lasting sleep/wake impairments, in that despite 4 weeks recovery sleep, CSS mice had less sleep during the normal sleep period (lights-on) and less wake in the active period (dark period). This pattern is consistent with sleep patterns observed at later stages in life, supporting the concept that CSS may injure the brain, at least in part, by aging aspects of brain function. In support of aging of the brain, we examined sirtuins. SirT1 and SirT3 decline in the brain with aging, where reductions with age contribute to age-related degeneration (57–59). LC and orexinergic neurons evidenced reductions in SirT1 and SirT3 and had increased lipofuscin, and these age-dependent effects also persisted at least 1 month into recovery, suggesting heightened vulnerability for a long time after CSS (36). The injury to LCn may not only affect vigilance but may also affect brain health, including the temporal progression of Alzheimer’s disease (AD). Specifically, in mouse models of AD, LC neuron lesions increase plaque burden, cognitive impairments, and tau hyperphosphorylation, while replenishing norepinephrine lessens deficits (27, 29, 60–62). In summary, CSS induces degeneration in select populations of neurons, including LC and orexinergic neurons, reduces sirtuins in the remaining wake-activated neurons and imparts a lasting disruption in sleep/wake patterns, supporting the concept that chronic insufficient sleep has lasting effects on brain health and function.

### Locus Coeruleus Response to Unihemispheric Sleep

A diverse collection of animal species demonstrate unihemispheric sleep, including aquatic mammals such as dolphin, seals, and sea lions, and many avian species. Fur seals can switch between bilateral hemispheric sleep when on land to unihemispheric sleep for extended periods when at sea (63). Presumably, unihemispheric sleep allows the animal motor activity to both keep the animal’s nose at the water’s surface and to maintain body temperature with muscle activity. Interestingly, during unihemispheric sleep, norepinephrine delivery to the cortex (from locus coeruleus) is reduced in both hemispheres (63). Similar responses are observed for two other wake monoaminergic neurotransmitters, histamine and serotonin. Thus, unihemispheric sleeping animals may have less demand on monoaminergic wake-active neurons across unihemispheric wakefulness. We do not know what happens to norepinephrine levels in the brain across sleep deprivation in unihemispheric sleepers, but it would be of great interest, as unihemispheric sleep deprivation leads to rebound only in the deprived hemisphere (64).

## Chronic Sleep Restriction Effects on Vigilance in Animal Models

Chronic sleep restriction in rats has been performed to determine whether psychomotor vigilance is impaired after CSS in rats (38), as it is in humans. Unlike humans, the rats do not show progressive or cumulative declines in performance across sleep restriction days, and unlike mice and humans the recovery is complete after one 24-h recovery period. However, the model implemented here is distinct from the above extended wake studies in mice and in humans. For these studies, rats were placed in a rotating drum for enforced ambulation continuously for 3 h, and then, they are provided a rest period of 1 h to eat, drink, and have a rest opportunity. Thus, this is not a model of extended wakefulness for long periods of time, and like the short-term sleep loss studies above for LCn, it is possible that the 3 h duration increases antioxidants to protect the neurons. Remarkably, performance was most impaired on the second day of sleep restriction, and by the third or fourth day of sleep restriction, vigilance improved (38). In humans, it is not the amount of sleep lost but the duration of wakefulness that predicts impaired PVT performance (2). Leemburg et al. performed a rigorous study of chronic sleep restriction in which rats were allowed 4 h sleep at the beginning of the lights-on period followed by complete sleep deprivation for 20 h (39). In this paradigm, rats showed persistence in sleep homeostasis in multiple parameters including shortened sleep latency, increased sleep attempts, and increased slow-wave activity (39). In contrast, there was no evidence for allostasis to CSS (39). Vigilance tests and neuronal counts were not performed in these animals. Further studies are needed to determine whether rats confer resistance to CSS-induced vigilance impairments; whether brief nap opportunities across extended wake can mitigate wake neuron injury; and whether the pattern of intermittent brief naps enables adaptive responses that protect the brain across future longer sleep loss exposures.

## Similarities and Differences with Effects of Sleep Fragmentation vs CSS

Many individuals with sleep disorders, including sleep apnea and periodic limb movement disorder, regularly experience fragmentation of sleep. These individuals may have normal total sleep durations for 24 h, but periods of sleep are frequently disrupted. This frequent disruption has been modeled in adult mice and shown to induce lasting neuronal injury (65). As with CSS, there are several unique models of sleep fragmentation. One method implements a rotor shaker table (65). Mouse cages may be placed on the rotor platform that is then gently moved at intervals around the clock, and this has been shown to effectively fragment sleep, while allowing group housing and normal eating patterns (65). In a recent study, group housed adult male mice were exposed to this form of sleep fragmentation for 14 weeks continuously (40). The animals’ weights were similar across sleep fragmented and control mice. The effects of sleep fragmentation on sleep-wake activity appear distinct from CSS. While CSS blunted the diurnal rhythm of sleep, sleep fragmentation markedly reduced wakefulness within the first few hours of the dark period, the most active period. Like CSS, sleep fragmentation resulted in a loss of both LCn and orexinergic neurons, although effects on LC were more pronounced (50 vs 25%) (40). Sleep fragmentation also increased oxidative stress in LCn and reduced SirT3. One of the most intriguing findings was that TNF-α was upregulated LCn, not in microglia (40). Gozal and colleagues have examined the effects of chronic sleep fragmentation on hypothalamic function in mice (41). They used a singly housed cage with a sweeper bar that intermittently sweeps the entire cage prompting the mouse to wake and move. With this paradigm, his group has identified leptin resistance, which may contribute to increased obesity with sleep fragmentation (41). His groups has also found that chronic sleep fragmentation induces lasting spatial (hippocampal-dependent) learning and memory impairments that require augmentation of TNF-α signaling and activated of NADPH oxidase (42, 43). Whether the TNF-α and oxidative stress changes are upstream, downstream, or independent of sirtuin changes should now be determined to elucidate effective therapies to prevent cognitive impairments and neural injury with chronic sleep disruption.

## Morphometric Effects of Chronic Sleep Loss

Several studies have now examined the ultrastructure of neurons in select brain regions in response to sleep loss. In an earlier study, the effect of 12, 24, and 36 h of total sleep deprivation on neuron ultrastructure was examined in four brain regions: the limbic cortex, the CA1 region of the hippocampus, the pontine reticular formation, and the locus coeruleus (44). At 12 h, all regions showed increased neurons with reparative changes (increased mitochondria, endoplasmic reticulum, lysosomes, chromatin, and nuclear invaginations). No degenerative changes were observed. At 24 h, greater percentages of neurons showed reparative changes in all four regions, and at 36 h, degenerative changes were observed in all regions. These degenerative changes included chromatolysis, somatodendritic vacuolization, and reductions in well-formed organelles. Intriguingly, layers III/IV of the limbic cortex and the CA1 region of the hippocampus showed greater percentages of injured neurons than the locus coeruleus. This work provides strong support that neurons are structurally altered differentially across subsets and by sleep loss duration. A recent study examined the effects of 2 months of sleep restriction to 4 h/day on the ultrastructure of cortical neurons (45). Although results were not quantified, the researchers concluded that there were more damaged mitochondria with missing or swollen cristae and swelling between the inner and outer membrane (45). Another recent study examined the effects of 4 days of sleep restriction on the ultrastructure of pyramidal neurons in the frontal cortex in young mice (4 weeks old) (46). CSS in this model was performed using a combination of approaches including an enriched environment and caffeine administration. Total daytime sleep time was reduced from 8.5 to 2 h for the 4 days. This study performed rigorous measures of neuronal mitochondria, finding larger mitochondria with CSS but no change in cristae or membranes to suggest injury (46). Because this study involved younger mice, a different sleep protocol and examined a different area than those above, it is not clear whether younger mice are less susceptible to CSS effects on neuronal mitochondria, whether the frontal cortex is less susceptible, or whether total sleep deprivation (longer extended wakefulness) provides a unique stress relative to CSS. CSS in these young rats does, however, increased lysosomes, consistent with above studies. In summary, both total and chronic partial sleep loss affect specific neuronal morphometrics, consistent with injury, and there is a sleep loss duration dose response for neuronal morphometric changes for severity of sleep loss. Both mitochondria and lysosomes seem particularly affected in most models.

## Neural Consequences of Sleep Loss During Brain Development

Brain circuitry is most plastic during development. Interestingly, brain development occurs at a time when animals, including humans, experience large proportions of sleep time in rapid eye movement (REM) sleep. If sleep, particularly REMS, impacts plasticity, then effects should be most pronounced across the most active periods of brain development, and indeed the work of several research groups supports this prediction.

Monocular visual deprivation during the critical developmental window in rat pups dramatically enhances synaptic strength within the relevant visual circuitry, dramatically remodeling the visual cortex for life (66). In contrast, little effect is appreciated in the adult rat. Strikingly REMS deprivation within this critical developmental window profoundly affects the window of visual plasticity. Specifically, 1 week of REMS deprivation extends the critical window for visual plasticity (47). In addition to REMS influencing the temporal course of the critical window for plasticity, spontaneous NREMS immediately after monocular deprivation enhances plasticity in the kitten, while sleep loss across this same time frame prevents plasticity (ocular dominance) (67). While molecular mechanisms underlying the effects of sleep on visual plasticity during development are not known, the process requires neurotrophins and protein synthesis (68). In summary, both REM and NREM influence brain plasticity in development.

Persistence of neurobehavioral deficits following sleep loss during the critical developmental window comes from work with *Drosophila melanogaster*. When flies are deprived of sleep as newly eclosed flies (first day beyond the larval stage), young flies have memory impairments that persist at least 6 days (48). In contrast, adult flies, losing the same total amount of sleep had learning impairments that are readily reversed with a few hours of recovery sleep (48). These key findings were extended by Kayser and colleagues (49) who demonstrated that sleep within a critical developmental window for the fruit fly is essential for the structural development of olfactory neuronal circuits involved in courtship behavior, and that sleep loss at day 1 post-eclosure results in lasting diminution of a specific olfactory glomerulus. Intriguingly, as the particular glomerulus affected by sleep loss grows rapidly, the metabolic status or growth history may predict vulnerability of a particular circuit.

## Effects of Sleep Loss on Neurogenesis

Across most of the adult lifespan, new neurons and glia are produced in specific brain regions. The production of new neurons (or neurogenesis) can be influenced by modifying health and lifestyle, including sleep time. Guzman-Marin et al. discovered that long-term sleep deprivation in adult rats (across 96 h) significantly suppressed the production of new progenitor cells in the hippocampus, relative to control animals and that this occurred without raising corticosterone levels (30). Moreover, adrenalectomized rats, lacking corticosterone, also demonstrate sleep deprivation suppression of neurogenesis (69). While the effects of sleep loss on neurogenesis are independent of corticosterone, sleep loss suppression of neurogenesis may require interleukin-1β (IL-1β). Specifically, mice lacking receptors for IL-1β do not reduce neurogenesis in response to sleep loss (70). Whether this is a direct or indirect effect (lifelong loss of a cytokine) has not been ascertained. In a follow-up experiment, the Guzman-Marin et al. group established that sleep loss specifically affected the generation of new mature neurons (71). A similar effect on neurogenesis is observed with sleep fragmentation where both 4 and 7 days of continuous sleep fragmentation suppressed neurogenesis, while 1 day did not (72). Neurogenesis is critical for optimal hippocampal learning and memory, and 3 days of sleep restriction (sleep loss during the first 6 h of the lights-on period) reduces the adult neurogenesis response to learning (73).

Intriguingly, an opposite effect of sleep loss on neurogenesis is observed for short-term sleep loss. Loss of one 12-h period of sleep actually increases both brain-derived neurotrophic factor (BDNF) and hippocampal neurogenesis and the increase in BDNF and neurogenesis are sustained for at least 1 month (74, 75). Junek et al. substantiated a time-dependent factor for sleep loss duration on hippocampal cell proliferation, finding no effect for 6 h sleep loss, while 12 h did indeed increase neurogenesis, while longer durations of sleep loss suppressed cell cycle cell proliferation (76).

## Effects of Sleep Loss on Neuroinflammation

There are clear bidirectional influences between sleep and the immune system (77). Yet, little is known of how chronic sleep restriction affects the neuroinflammatory responses. Most of the studies have examined acute (short-term) sleep loss or chronic sleep fragmentation. Specifically, short-term sleep deprivation can suppress the peripheral immune responses to vaccination, and sleep disruption increases TNF-α, IL-1β, and interleukin-6 in specific brain regions and peripheral tissues (78–80). Total sleep loss for 24 h increases IL-1β in the hypothalamus and brainstem twofold (81). Mice exposed to chronic sleep fragmentation, but not reduced total sleep, develop increased TNF-α in cortical tissue (42). The increased TNF-α may contribute to sleepiness from sleep fragmentation, in that the sleepiness can be prevented in mice lacking TNF-α receptors (31). Acute sleep loss can also upregulate TNF-α within neurons (40). This may be in response to persistent neuronal activation, as whisker stimulation increases TNF-α in neurons in activated neurons in the barrel cortex (82, 83). Cytokine signaling in neurons can modify hypocretin signaling by reducing pre-pro-hypocretin and orexin receptor 2 mRNA (84). At the same time reducing central TNF-α signaling can reduce sleep, particularly slow-wave sleep (85), and IL-1β injected into the sleep-promoting preoptic area of the hypothalamus and wake-activating basal forebrain, increases the firing of sleep-active neurons and reduces the firing of wake-active neurons. Major questions prompted by the above findings include (1) What is the origin of sleep loss cytokine responses (neuronal or glial or other)? (2) How does neuronal activation increase intra-neuronal TNF-a and is this upregulation of consequence in the neuron? and (3) can suppression of cytokine responses hasten neurobehavioral recovery from sleep loss and/or prevent neuron loss?

## Effects of Sleep Loss on Non-Neuronal Brain Cells

**Figure 1:**
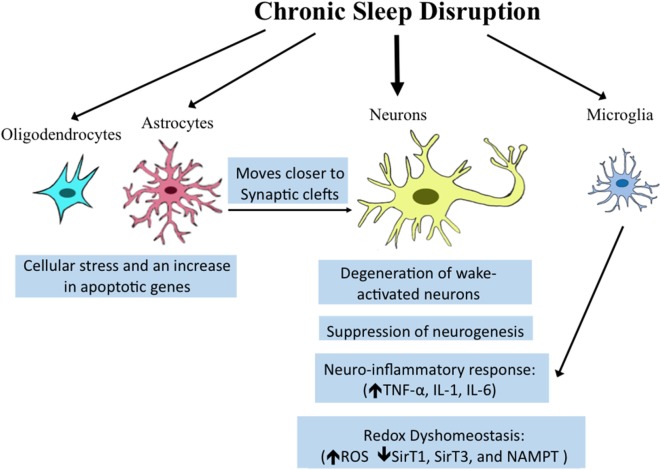
**Overview of reported effects of sleep/wake disturbances on neural cells**. Chronic sleep disruption which includes chronic total and partial sleep deprivation, as well as sleep fragmentation, can influence diverse brain cell types. Established molecular and morphologic responses within specific cell types are highlighted in blue boxes below the various cell types. Microglia share a pro-inflammatory response, also observed in neurons in resonse to sleep loss. The effects of sleep disruption on astrocytes on synaptic clefts may influence neuronal synapse function.

Once believed to be simply structural components of the brain, non-neuronal cells, including microglia, astrocytes, and oligodendrocytes play critical roles in synaptic maintenance, energy substrate delivery, and clearance of waste (86–89). Recent studies provide evidence that transcriptional regulation in both oligodendrocytes and astrocytes is influenced by behavioral state. Spontaneous sleep and wake differentially effect transcription profiles for enriched oligodendrocytes pooled from behaviorally monitored adult mice (90). Importantly, spontaneous wakefulness increases genes involved in cellular stress processes, including apoptosis genes (90). Intriguingly, optogenetic stimulation of astrocytes within the posterior hypothalamus at one specific frequency (10 Hz) increases both NREMS and REMS (91). How specifically this occurs and what messages are conveyed from astrocytes to neurons are not yet understood. Both spontaneous wake and enforced short-term wake reduce the number of oligodendrocyte progenitor cells (90), but whether total numbers and functionality of oligodendrocytes are compromised with extended wake has not been explored. Astrocytes demonstrate more pronounced responses to wake than oligodendrocytes with sevenfold more genes upregulated in wake than in sleep (50). Intriguingly, many of the genes upregulated are related to metabolism and/or energetics. Astrocytes release lactate as an energy substrate for neurons (92), and this is regulated in a wake-dependent fashion (93). In addition to changes in astrocyte genes related to metabolism, changes were observed for genes related to astrocyte process extension. The morphology of astrocytes has been examined with electron microscopy, showing that in response to CSS, astrocytic processes moves closer to synaptic clefts and this is not only wake-activated but activity specific so that regions of the brain activated by a given stimulus in waking show greater responses. However, because astrocytes were not marked specifically, it is possible that the processes examined included microglial processes. The collective neuronal and glial responses are summarized in Figure 1.

## Effects of Sleep Loss on Hippocampal Function and Connectivity

An elegant body of research demonstrates a critical role for sleep in learning in the immediate period after contextual training. This hippocampal-dependent form of learning can be attenuated by sleep loss in a window of 5–6 h after training, and this effect from sleep loss on diminishing recall is not secondary to learning other material while wake (interference learning) (32, 94, 95). This vital role played by sleep has been systematically elucidated largely by the work of the Ted Abel lab. Short-term sleep loss in the window after training reduces cAMP in hippocampal neurons, leading to reduced protein kinase A and less phosphorylation of CREB for its activation (94) Activation of CREB is essential for the memory consolidation (96). This in turn prevents mTORC1-dependent protein translation for memory consolidation (97).

Less is known of how CSS affects hippocampal function and health. A recent study in humans examined hippocampal and thalamic gray matter volume by MRI before and after 72 h total sleep deprivation (98). The group found reduced volume in the thalamus but not in the hippocampus. There is, however, a differential susceptibility to impaired hippocampal function upon sleep loss, while hippocampal structure can predict the extent of impairment in hippocampal function following sleep loss (99). Whether sleep loss changes in synaptic densities or neuronal function occur in humans remains an open question, but this has been examined in wild-type adult male mice (100). Intriguingly, the loss of spines was regionally specific (in CA1 and not CA3) and dependent upon upregulation of cofilin, a protein that degrades actin filaments, structural elements of dendritic spines. This intriguing finding suggests that short-term sleep loss impairs memory, in part by degrading synapses and supports the concept that specific synapses are either lost or reinforced in sleep (101–104). In summary, short-term sleep loss reduces dendritic spines and therefore connectivity in specific regions of the hippocampus, and loss of spines predicts learning impairment for hippocampal-dependent learning and memory. Whether CSS acts in a similar way and whether effects are reversible with recovery sleep should be explored.

## Concluding Remarks

The importance of sleep within an acute window in learning and memory is now firmly established, as is the need for adequate sleep long term for peripheral metabolic homeostasis and cardiovascular health. The concept that chronic sleep loss may have lasting or even protracted recovery effects on brain function has only recently begun to be substantiated, where it is now evident that chronic sleep loss can have profound effects on brain health and function, including neuronal survival. CSS that occurs within the temporal window of brain development imparts lasting disturbances in behaviors critical to the perpetuation of a species. Select populations of wake-activated neurons are highly susceptible to degeneration in conditions of CSS in young adult mice, where extended wakefulness results in mitochondrial metabolic and inflammatory stress, in part, by reducing sirtuins and increasing cytokine responses, respectively, in vulnerable neurons and/or brain regions. Neurons are not the only brain cells affected by sleep loss. Significant changes occur in oligodendrocytes, astrocytes and microglia. Molecular pathways involved are beginning to be understood, providing novel pharmacotherapeutic avenues to protect the brain across sleep loss. Collectively, this body of research on CSS emphasizes the need to develop therapies to prevent neural injury in the common practice of CSS.

## Author Contributions

SV, ZZ, and XZ all identified relevant literature, read the body of work related to the topic, discussed format, wrote sections, and edited others. ZZ made the figure.

## Conflict of Interest Statement

The authors declare that the research was conducted in the absence of any commercial or financial relationships that could be construed as a potential conflict of interest.
